# Checkpoint inhibition immunotherapy for advanced local and systemic conjunctival melanoma: a clinical case series

**DOI:** 10.1186/s40425-019-0555-7

**Published:** 2019-03-25

**Authors:** Paul T. Finger, Anna C. Pavlick

**Affiliations:** 1 The New York Eye Cancer Center, Suites 5A/B, 115 East 61st Street, New York, NY 10065 USA; 2 New York University Cancer Institute, 160 East 34th Street, New York, NY 10016 USA

**Keywords:** Checkpoint, Immunotherapy, Melanoma, Conjunctiva, Metastasis, Ipilimumab, Pembrolizumab, Nivolumab, Topical, Interferon

## Abstract

**Background:**

Herein, we describe the use of systemic immunotherapy for both locally advanced and metastatic conjunctival melanoma. Current treatments for advanced conjunctival melanoma typically result in poor local control leading to disfiguring orbital exenteration surgery. Locoregional spread of conjunctival malignant melanoma typically requires pre-auricular and cervical lymph node dissection with post-operative adjuvant radiation therapy. In addition, classic systemic chemotherapy has been unsuccessful in the treatment of metastatic disease.

**Methods:**

This is a retrospectively analyzed clinical case series of 5 patients with biopsy proven conjunctival melanoma who were treated with checkpoint inhibition therapy. Of these, 3 patients were treated for residual ocular disease present after failing multiple local therapies and refusing orbital exenteration surgery and two (with local ocular control) for metastatic conjunctival melanoma. Both those with locally advanced disease and patients with metastatic disease received an anti-PD1 agent in combination with another immunotherapeutic agent. All 5 were given multiple cycles of systemic anti-PD1 therapy, 1 was initially treated with single agent ipilimumab (3 mg/kg) prior to approval of anti-PD1 agents and two received interferon eye drops. As part of each ophthalmic examination, photographs of all conjunctival and eyelid surfaces were obtained. Systemic evaluations involved initial staging scans as well as periodic re-imaging.

**Results:**

All cases have shown responses. Of the 2 complete responses, 1 was a patient with systemic disease. No patients developed ocular toxicity or loss of vision. However, systemic adverse effects included adrenal insufficiency, Grade-III colitis, Grade-II dermatitis, Grade-II hepatotoxicity and Grade-II pneumonitis.

**Conclusions:**

This report suggests that systemic immunotherapy with or without topical interferon is effective in treatment of malignant melanoma of the conjunctiva. Therefore, it can be considered for patients with advanced local conjunctival melanoma, those who refuse orbital exenteration surgery and those with systemic metastasis.

## Introduction

Conjunctival malignant melanoma (CMM) is epidemiologically, molecularly and genetically different from intraocular “uveal” melanoma [1]. Unlike intraocular melanomas, CMM commonly present in the sun-exposed, interpalpebral, epibulbar surface. Incidence in the United States and Finland is reportedly increasing [2, 3].

More closely related to mucosal melanomas, early stage CMM tend to start as localized, “in situ,” relatively flat and superficial spreading tumors of the epithelium [1, 4]. They commonly extend onto the corneal epithelium and rarely invade the eye. In contrast, late-stage CMM tend to be multifocal with skip and amelanotic tumors, exhibiting high post-treatment recurrence rates [5, 6]. Conjunctival melanomas eventually grow in a vertical phase, both invading beneath the conjunctival epithelium and develop nodules [1, 4]. Metastasis to both regional pre-auricular and cervical lymph nodes is typically followed by more widespread systemic disease [4, 5]. CMM can also spread into the orbit, eyelids, sinuses and brain.

The American Joint Committee on Cancer (AJCC) staging system utilizes tumor size and location CMM at presentation to predict local control and metastasis [4]. For example, up to 88% local control of localized primarily epibulbar tumors has been achieved with local excision aided by double freeze-thaw cryotherapy [5–8]. However, large, diffuse and multifocal CMM have been associated with up to 50% local recurrence rates [7]. From Seregard’s survey of 21 reports on a cumulative 1273 patients, there was a mean 29.2% (range 9–61%) incidence of CMM metastasis [1].

In an effort to improve local control, topical chemotherapy eye drops (ie: mitomycin, interferon) have been used for CMM [7–9]. Proponents of topical chemotherapy note that it addresses diffuse, multifocal and non-pigmented subclinical disease. While topical interferon is extremely well tolerated, side effects associated with topical mitomycin include: conjunctivitis and blepharitis, keratitis and puntal stenosis [7–9].

In practice, combinations of surgical excision, cryotherapy and topical chemotherapy have been effective for local control of T1N0M0 and T2N0M0 staged disease. However, these modalities can cause tear dysfunction and scarring with loss of vision. In cases of recurrence, patients often face repeated surgeries, where the eyelids can become attached to the globe, tethering eye movement and cause corneal exposure as well as obscuring conjunctival surfaces from further examination. For locally advanced AJCC-T3N0M0 and T4N0M0 CMM, orbital exenteration (removal of the eye, lids and orbit) with adjuvant orbital radiation therapy may be the only way to completely remove or destroy the ocular CMM [10, 11]. Sagiv et al. have recently reported successful anti-PD1 immunotherapy for 5 patients with metastatic conjunctival melanoma with up to 36 months follow up [12]. Herein, we present a clinical case series where systemic immunotherapy therapy was employed for treatment of locally advanced and metastatic CMM.

## Methods

### Examinations

Ophthalmic examinations included: a medical history, visual acuity measurement, ocular-motor evaluation, visual field assessment, slit-lamp examination with observations of all conjunctival surfaces (with eyelid eversion), palpation of pre-auricular and cervical lymph nodes and funduscopic (retinal and optic nerve) examination. High-frequency ultrasound imaging was used to evaluate the conjunctival tumor and for extension into the subjacent sclera, uvea and anterior chamber.

Systemic CMM staging included PET/CT or CT scans [13]. The 3 patients with tumors that would require orbital exenteration for complete removal underwent a detailed discussion of the potential risks and benefits of observation, radiation (electron, proton, photon), excision with or without cryotherapy (with or without topical chemotherapy) and exenteration surgery with post-resection radiation therapy. The 2 patients with locally controlled ocular CMM, discovered to have metastasis by periodic surveillance, were directly offered systemic treatment.

### Treatment rationale

Two of the three patients with locally advanced disease (ages 76–94) were initially treated with single agent pembrolizumab, since adverse effects with anti-PD-1 therapy are approximately about 10% and it generally well tolerated. One patient received single agent ipilimumab initially, since anti-PD1 therapy had not been Food and Drug Administration (FDA) approved. Despite an older age, all patients had an ECOG performance status of 0–1.

After at least 3 months of single agent therapy, if no clinical response was seen, low dose ipilimumab (1 mg/kg) every 3 weeks × 4 doses or interferon eye drops were added. Our unique rationale for adding a second immunotherapeutic agent was either to try to increase PD-L1 expression on the tumor, thus making it more responsive to anti-PD-1(programmed cell death protein-1) therapy or with topical interferon eye drops by triggering a local inflammatory reaction trying to enhance cytotoxicity and survival of NK cells, induce the generation and survival of both cytotoxic T-lymphocytes (CTL) and memory cluster of differentiation (CD8+) T-cells, promote CD8+ T-cell priming against tumor antigens and anti-angiogenic effects on tumor vasculature [14, 15].

The two patients with diffuse metastatic disease were treated with immunotherapy. One patient was treated sequentially, since anti-PD1 was not FDA approved at the time of her initial metastatic presentation, however, the second patient received combination therapy ipilimumab/nivolumab regimen due to the vast extent of her disease, rapidity of onset and overall otherwise excellent physical condition.

## Results

### Systemic treatment for advanced local conjunctival melanoma

#### Case #1

**Fig. 1:**
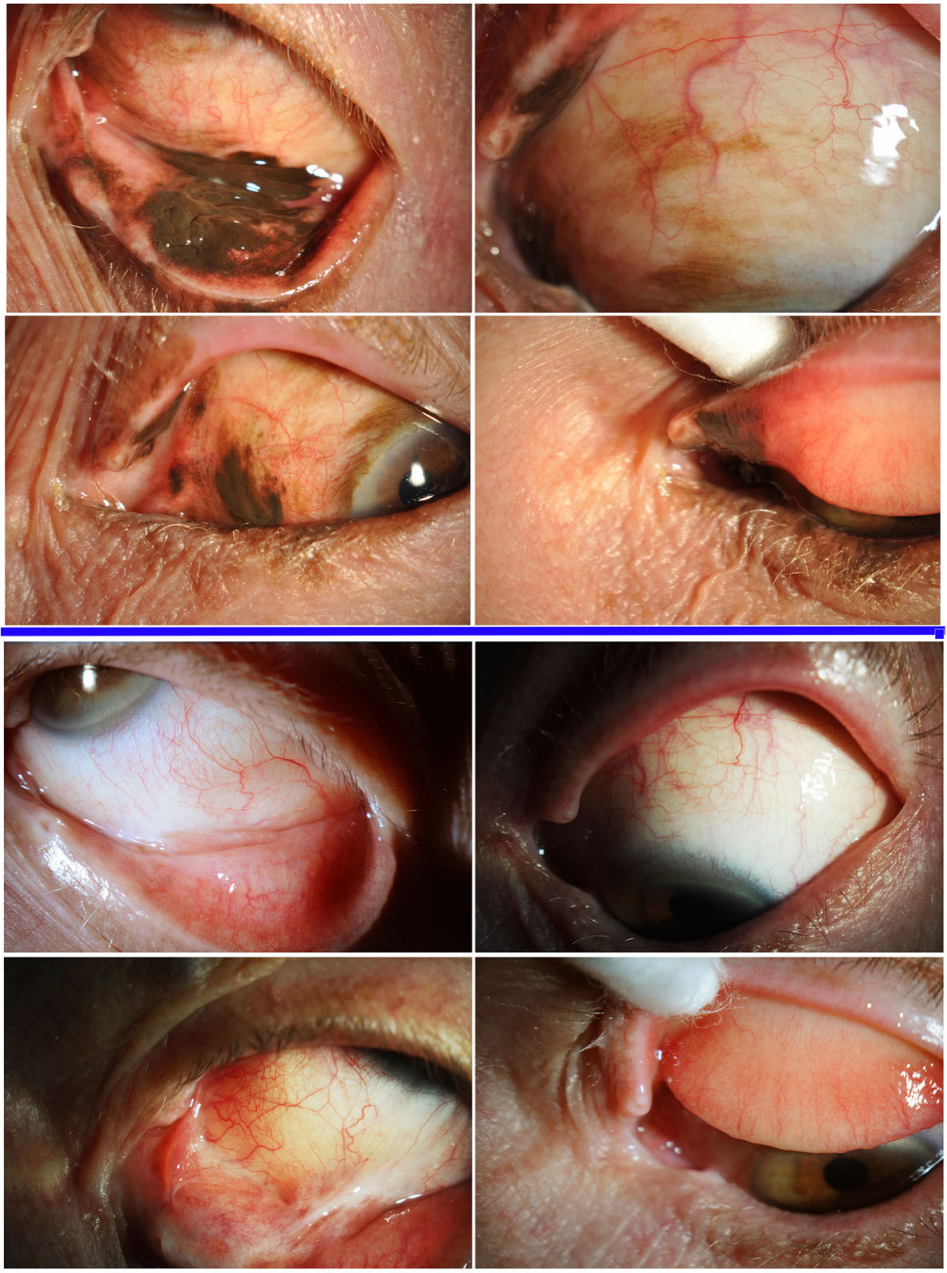
Top, 4 photographic images demonstrating the extent of local bulbar and palpebral conjunctival melanoma prior to systemic immunotherapy. Bottom 3 years later, 4-photographic images demonstrate the extent of regression after combination systemic and topical chemotherapy. Please note that the top 4 images (below the blue line) correspond to the bottom 4 images as before and after photographs. For example, the top left image, of the top 4 images corresponds to the top left of the bottom 4 images

An otherwise healthy 76-year-old male had a history of multiple, failed local treatments for conjunctival melanoma since 1987. He sought second opinion due to worsening conjunctival pigmentation with tumor-extension onto the eyelid skin despite 6-months of topical interferon-alpha chemotherapy (Fig. 1, top) [8].

Ophthalmic examination revealed 20/20 vision, diffuse, multifocal CMM extended onto the cornea, caruncle and eyelid skin (AJCC-T3bN0M0). No regional lymphadenopathy was palpable. High-frequency ultrasound imaging and ophthalmoscopy showed no intraocular invasion. PET/CT revealed no regional or systemic metastasis.

In consideration of systemic immunotherapy as an alternative to standard therapy, genetic and molecular markers were obtained by biopsy. This revealed the tumor was BRAF,c-Kit, NRAS wild-type. After informed consent, he was treated with single agent, ipilimumab (anti-CTLA4 monoclonal antibody) in February 2015. After 3 cycles of therapy and no visible tumor-response, he developed autoimmune drug induced adrenal insufficiency. The ipilimumab was discontinued and an endocrinologist prescribed steroid replacement.

His conjunctival melanoma continued to progress. Therefore, we started pembrolizumab 2 mg/kg every 3 weeks in August 2015. There was minimal response until interferon alpha eye drops (1 million units/cc, 4 times daily which appeared to synergize anti-PD1 therapy) was added. This combination treatment regimen was continued until February 2017, when pembrolizumab was stopped due to a near complete clinical response.

It is important to note that he developed Grade-II dermatitis (pruritis and rash) during this treatment which was managed with topical corticosteroid cream and oral anti-histamines. He has remained off pembrolizumab until February 2017, but continued the same dose of topical interferon eye drops applied to the eye lid. At last follow up, (36-months after initiation of therapy and 2 years NED) the conjunctival tumor had completely resolved. Only a small area of cutaneous hyper-pigmentation can be seen on the lower eyelid (Fig. 1, bottom).

#### Case #2

A 94-year-old female presented with a CMM [cT3bN0M0, pT4b (greater than 4.0-mm thickness with ulceration)]. Ophthalmic examination revealed 20/25 vision and CMM involving most of the epibulbar conjunctiva, superior tarsus, caruncle and eyelid skin. No regional lymphadenopathy was palpable. Ultrasound imaging revealed no evidence of intraocular invasion.

In consideration of her age, comorbidities and refusal of orbital exenteration surgery; systemic treatment was recommended. In March 2017, the patient started treatment with single agent intravenous (IV) pembrolizumab 200 mg every 3 weeks. After 3 cycles, topical interferon eye drops (1 million units/cc four times a day) were recommended but refused. After 4 cycles of pembrolizumab, progression was marked by an increase in thickness of two epibulbar nodules.

In July 2017, ipilimumab 1 mg/kg IV was added to the pembrolizumab 200 mg IV every 3 weeks. She received a total of 4 cycles of combination low dose ipilimumab with pembrolizumab, which was associated with partial clinical regression and no toxicity. Unfortunately, her systemic therapy has to be discontinued due to advancing, non-therapy related, congestive heart failure from which she died 5 months later.

#### Case #3

An 84-year-old female with a long-standing history of CMM had received multiple local therapies including: excision, cryotherapy, topical mitomycin and eye plaque brachytherapy for recurrent CMM. Her ophthalmologist recommended orbital exenteration, after which she sought alternative treatment.

Due to prior treatments, her eyelids were dysmorphic and nodular. The conjunctival fornices were shortened causing persistent corneal exposure and scarring. Ophthalmic examination revealed hand motions vision. The CMM involved the entire conjunctiva and most of the corneal surface. It extended onto and through the upper and lower eyelids. No regional lymphadenopathy was palpable. By PET/CT, she was staged AJCC-T3bN0M0.

Pembrolizumab 200 mg IV every 3 weeks was started in August 2017 with minimal improvement and no adverse events (after 4 cycles of therapy). Low dose ipilimumab (1 mg/kg IV every 3 weeks) was added to pembrolizumab in November 2017. After 4 doses of combination therapy there was a 50% local tumor reduction. Unfortunately, while on hiatus from immunotherapy for a cataract extraction, new pigmented tumor nodules recurred on her upper and lower eyelids. One year after her first treatment (November 2018), she was restarted on a combination of monthly intralesional interferon alpha (3 million units per eyelid) in combination with restarting ipilimumab and pembrolizumab and is alive at her last visit (February 2019).

### Systemic treatment for metastatic conjunctival melanoma

#### Case #4

A 76-year-old female presented with an NRAS (Q61R) mutated, CMM of the left eye in July 2009. In March 2010, she underwent successful eradication of her local CMM utilizing a combination of excision, cryotherapy and topical mitomycin chemotherapy. However, as a result of treatment she suffered significant corneal toxicity which required two amniotic membrane implants as well as an autologous corneal stem-cell transplant to stabilize.

In February of 2011, she developed a mass in her left parotid gland. A parotidectomy, facial nerve dissection and suprahyoid neck dissection revealed regional spread of her CMM. Surgery was followed by adjuvant regional radiation therapy. Then, in January 2012, surveillance computed tomography of the lungs revealed mediastinal lymphadenopathy. Bronchoscopy with mediastinoscopy revealed 2/6 level II LN +, 2/4 level IV LN + melanoma metastases.

Treatment involved mediastinal-irradiation, followed by immunotherapy. Four induction cycles of ipilimumab (3 mg/kg IV every 3 weeks × 4 doses) were tolerated without adverse effects. Then, in November of 2013, she developed a new subcarinal metastasis. Treatment consisted of video-assisted thoracoscopic surgical resection of this nodal mass, followed by adjuvant radiation therapy and concurrent ipilimumab (3 mg/kg IV every 3 weeks × 4 doses).

Three years later (a longer hiatus as compared to her prior episodes of recurrence may have been attributable to her prior immunotherapy), she was noted to develop a left buttock subcutaneous nodule (October of 2014). The nodule was resected and she was observed, until the nodule recurred in her left buttock in February 2015. The solitary nodule was re-excised and she was treated with post-excision adjuvant buttock radiation (5000 cGy in 20-fractions) together with pembrolizumab 200 mg IV every 3 weeks for 2 doses followed by single agent pembrolizumab 200 mg every 3 weeks for 42 weeks (total adjuvant therapy lasting 6 months). After completing treatment in June 2016, she has remained NED for 2 years; alive to last follow up in January 2019.

#### Case # 5

A 72-year-old female with a BRAF V600K mutated, T1d epibulbar CMM (2.9-mm thick) was treated by local-excision with subsequent topical chemotherapy resulting in local control, vision and ocular preservation for 9 years.

However, in February 2016 she noticed a subcutaneous nodule on her right flank. A biopsy revealed metastatic CMM. Staging work up demonstrated metastatic disease in the lungs, liver, bone, nodes and multiple subcutaneous deposits.

She was started on systemic immunotherapy with intravenous ipilimumab 3 mg/kg with nivolumab 1 mg/kg IV every 3-weeks for an anticipated 4 total doses. She tolerated treatment for the first 2 cycles with resolution of all palpable subcutaneous nodules. However, she developed Grade-II hepatotoxicity which resolved with treatment delay. Following her third dose of combination therapy, she developed Grade-III colitis which was treated with intravenous fluids and corticosteroids. She had no improvement of her colitis, so, she received infliximab (5 mg/kg) IV. This resolved her diarrhea and she was slowly tapered off corticosteroids. She then developed a delayed onset Grade-II pneumonitis which resolved after a short course of oral corticosteroids and inhalers.

**Fig. 2:**
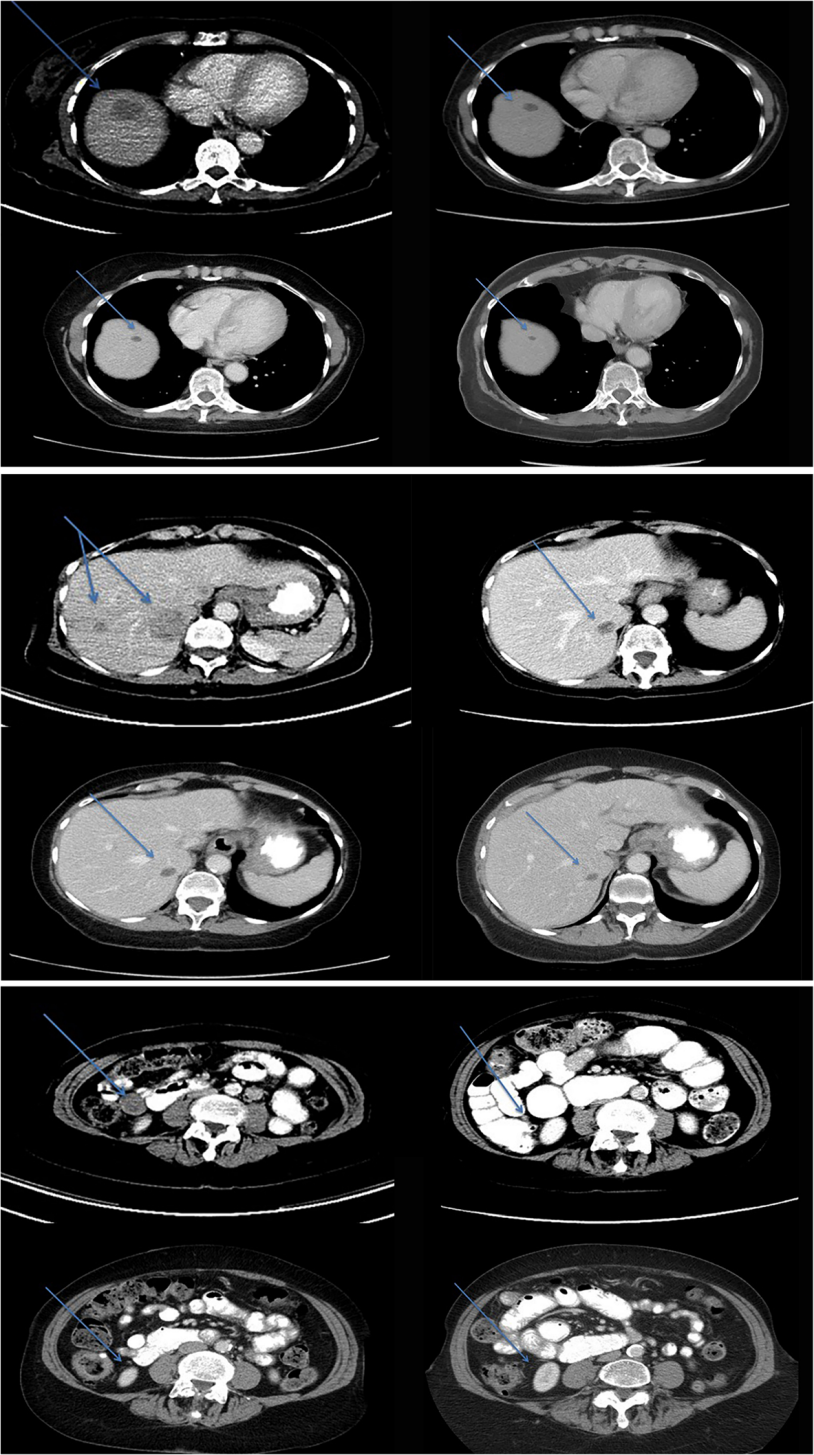
Top 4-up images, Patient #5, note the contrast enhanced, transverse abdominal computed tomography (CT) reveal a large solitary CMM metastasis on 2/2016, 7/2016, 4/2017 and 10/18 respectively (arrows). Middle 4-up CT-images reveal two additional CMM metastases on 2/2016, 7/2016, 2//2017 and 10/2018 respectively. Bottom 4-up CT images of a 2.7 mm abdominal implant prior to treatment on 2/2016, 7/2016, 4/2017 and 10/2018 where resolution is noted

Surveillance revealed a 75% reduction in systemic tumor burden in November 2016 and no evidence of systemic disease, determined to be NED for 3 years in October 2018 (Fig. 2). In this case, the patient had not received any further systemic therapy after the 3 induction doses of ipilimumab and nivolumab leading to a sustained durable remission.

## Discussion

Local and metastatic conjunctival melanoma responded to check-point inhibition immunotherapy. Complete responses were noted in one locally advanced and one metastatic CMM. In addition, there were 2 significant partial ocular responses. Immuno-modulating agents included systemic ipilimumab, nivolumab, pembrolizumab and topical interferon alpha. Our findings of regression indicate that immunotherapy may provide the best option for CMM patients who refuse exenteration surgery, are poor candidates for anesthesia and those with metastatic disease.

Cutaneous, choroidal and conjunctival melanomas have different mutational patterns [16–20]. The BRAF and NRAS proto-oncogene, GTPas genes associated with the MAPK pathway are present in cutaneous melanomas and up to 50% of conjunctival melanomas (unlike other mucosal and intraocular melanomas). Thus, B-Raf mutations and sun-exposure may link conjunctival melanomas and cutaneous melanomas. However, additional epidemiologic complexities exist. For example, a study of 53 patients showed a higher percentage of KIT than B-Raf mutations in Chinese patients with conjunctival melanoma. Patterns of metastatic spread are also similar in cutaneous and conjunctival melanomas (nodal, subcutaneous, lung and bone) compared to choroidal melanomas (predominantly hepatic). This shared mutational pathway also makes targeted agents potential therapeutic options for metastatic CMM patients.

In review of the published literature using the terms conjunctiva, melanoma, BRAF, KIT, pembrolizumab, ipilimumab, interferon and genetics, we found a 2016 study on the efficacy of anti-PD-1 agents in acral and mucosal melanomas supported its use in clinical practice [21, 22]. In contrast, a 2017 study concluded that checkpoint inhibition for advanced mucosal melanoma revealed no clinical response and disease progression [23]. However, in both studies, no conjunctival melanomas were mentioned.

This case series presents compelling evidence that conjunctival melanoma responds to immunotherapy. Our partial and complete (local and systemic) responses associated with systemic immunotherapy add 5 cases of advanced conjunctival melanoma that have responded to systemic immunotherapy [12]. It also demonstrated that checkpoint immunotherapy was well tolerated by the two (84 and 94-year-old) elderly patients; who were treated without any appreciable toxicity.

The weaknesses of our study rest in its retrospective design, clinically tailored treatment regimens and lack of long-term follow-up. However, we selected the most advanced cases, with large tumor burdens less likely to respond to therapy. In such advanced cases where local treatment (orbital exenteration) is disfiguring and questionably effective, systemic immunotherapy was found acceptable during informed consent.

In that most patients with locally advanced CMM are treated by orbital exenteration; the known side effects of checkpoint inhibitor systemic immunotherapy [i.e. fatigue, diarrhea, colitis, hepatitis, pneumonitis, endocrinopathy (thyroid dysfunction, hypophysitis, adrenal insufficiency, diabetes)] and less common side effects must disclosed and discussed during the shared decision making critical to informed consent. For patients with metastatic CMM; there is no effective alternative.

In conclusion, advanced CMM (local and systemic) was found to respond to systemic immunotherapy. Specifically, PD-1 inhibitors in conjunction with other biologic therapies (ipilimumab, topical interferon eye drops, and radiation therapy) achieved durable responses to treatment for locally advanced and metastatic CMM. Therefore, larger more significant investigations and/or a prospective data registry of treatment for advanced ocular disease and systemic metastasis are warranted.

## Abbreviations

AJCC: American Joint Committee on Cancer
CD8+: Cluster of differentiation 8 +
CMM: Conjunctival malignant melanoma
CT: Computed tomography
CTL: Cytotoxic T-lymphocytes
CTLA4: Cytotoxic T-lymphocyte Associated protein 4
FDA: Food and Drug Administration
IV: Intravenous
Kg: Kilogram
Mg: Milligram
NED: No Evidence of Disease
PD-1: Programmed cell death protein 1
PET/CT: Positron emission tomography / computed tomography
TNM: Tumor, Node, Metastasis
